# Cardio-renal benefits of sacubitril/valsartan in patients with advanced chronic kidney disease: experience in daily clinical practice

**DOI:** 10.1186/s12882-022-02919-z

**Published:** 2022-08-23

**Authors:** María Dolores Martínez-Esteban, Teresa Vázquez-Sánchez, Rafael Pozo-Álvarez, Alicia Moreno-Ortiz, Juana Alonso-Titos, Guillermo Martín-Reyes, Pedro Ruiz-Esteban, Daniel Gaitán-Román, Domingo Hernández

**Affiliations:** 1 Nephrology Department, Hospital Regional Universitario de Málaga, University of Málaga, Instituto de Investigación Biomédica de Málaga (IBIMA), REDinREN (RD16/0009/0006 and RICORS RD21/0005/0012), E-29010 Málaga, Spain; 2 Cardiology Department, Hospital Regional Universitario de Málaga, E-29010 Málaga, Spain

**Keywords:** Chronic kidney disease, Heart failure, Reduced ejection fraction, Neprilysin inhibitor, Glomerular filtration rate

## Abstract

**Background:**

The association between cardiac complications, such as heart failure (HF), and chronic kidney disease (CKD) is well known. In this study, we examined the effectiveness and safety of treatment with neprilysin inhibition in patients with advanced chronic kidney disease (stage 3b-4).

**Methods:**

This single-centre, longitudinal, retrospective study of 31 months duration involved consecutive patients with CKD and HF with a reduced ejection fraction (HFrEF) who started treatment with sacubitril/valsartan. Glomerular filtration rate (GFR), cardiovascular risk factors, proteinuria, potassium, echocardiographic parameters and admissions for heart failure were analysed.

**Results:**

The study comprised 25 patients with a median age of 73.2 ± 5.9 years. The most frequent aetiology of heart failure was ischemic heart disease. The median GFR was 29.4 ± 8.3 ml/min/1.73 m^2^ and the left ventricular ejection fraction (LVEF) 36.4 ± 8.9%. The GFR improved after initiating the treatment (F = 3.396, *p* = 0.019), as did the LVEF at one year of follow-up (*p* = 0.018). The number of visits to the emergency department for heart failure was also reduced. No patients needed to start renal replacement therapy.

**Conclusions:**

This study shows that sacubitril/valsartan may play a beneficial role in patients who have advanced CKD and HFrEF, with a satisfactory safety profile.

## Background

Cardiac complications are the main cause of death among chronic kidney disease (CKD) patients, and heart failure (HF) with a reduced ejection fraction is a major contributor in these patients to both this mortality and HF-associated hospitalization [1].

Natriuretic peptides have beneficial effects, including natriuresis, increased diuresis, vasodilatation and inhibition of the renin-angiotensin system (RAS). Neprilysin is the key enzyme responsible for degrading natriuretic peptides and other vasoactive substances like angiotensin II, bradykinin, endothelin, and substance P [2]. Compared with RAS blockers the angiotensin receptor-neprilysin inhibitor (NEPi) sacubitril/valsartan has demonstrated a reduction in both mortality and HF-related hospitalization in symptomatic patients with a reduced ejection fraction less than 35% [3–5]. Although inhibition of neprilysin raises the concentration of circulating natriuretic peptides, it also leads to reflex RAS activation and inhibits angiotensin II breakdown. Thus, NEPi must be combined with RAS blockers to achieve beneficial effects. The PARADIGM trial (prospective comparison of NEPi with angiotensin-converting enzyme inhibitors to determine the impact on global mortality and morbidity in heart failure) demonstrated that sacubitril/valsartan decreased the risk of cardiovascular mortality in patients with HF with a reduced ejection fraction compared to enalapril [3]. However, patients with CKD (GFR < 30 ml/min/1.73 m^2^) were excluded from the PARADIGM-HF trial. Cardiovascular disease and CKD frequently coexist and patients with severe CKD are at very high risk for cardiac events [6–10]. Thus, the efficacy of this medication in patients with more advanced CKD remains uncertain and data are lacking on tolerability and outcome of patients with severe CKD and HF with a decreased ejection fraction in the medium- and long-term.

The aim of this study, therefore, was to assess the efficacy of sacubitril/valsartan in daily clinical practice in patients with severe CKD and HF with a reduced ejection fraction. We analysed the evolution of the ejection fraction by sequential serial echocardiographic studies. Additionally, we assessed changes in GFR and proteinuria in patients under treatment with this medication for 24 months.

## Methods

This single-centre, longitudinal, retrospective study comprised consecutive patients treated with sacubitril/valsartan followed at the nephrology outpatient clinic who had started the treatment between October 2016 and January 2020.

The inclusion criteria were: age > 18 years, chronic symptomatic heart failure with a reduced left ventricular ejection fraction (LVEF) and New York Heart Association functional status II-IV, plus CKD with an estimated glomerular filtration rate (eGFR) of < 50 mL/min/1.73m2. The exclusion criteria consisted of: allergy to any of its components, hypotension (systolic blood pressure < 100 mmHg) or hyperkalaemia, defined as a baseline potassium concentration > 5.4 mEq/L. In all cases previous treatment with angiotensin receptor antagonists II (ARAII) and angiotensin converting enzyme inhibitors (ACEI) was suspended prior to starting sacubitril/valsartan, with a washout period of 36 h for ACEI. Other antihypertensive medication, including diuretics, was reduced with the start of sacubitril/valsartan. The initial dose was 24/26 mg each 12 h. If the potassium and blood pressure figures allowed, the dose was titrated to 49/51 mg each 12 h. Higher doses were not used in accordance with the technical specifications of the product.

Baseline data recorded included sex, age, cardiovascular risk factors (hypertension, diabetes mellitus and dyslipidaemia), and renal function, measured as serum creatinine (mg/dL) and GFR estimated with the CKD-EPI formula, serum potassium (mEq/L) and the urine protein:creatinine ratio (mg/g). Data were also recorded relating to heart disease aetiology, LVEF measured by echocardiography, medical treatment and visits to the emergency department with congestive symptoms. Follow-up data included the blood pressure figures, any possible side effects of the treatment or its cessation, and kidney function parameters. A control echocardiogram was done once yearly. The cause of death of the patient was recorded if this occurred.

All the data were obtained during the course of usual clinical practice. The study was undertaken in accordance with the norms of good clinical practice and the ethical concepts of the Declaration of Helsinki.

### Statistical analysis

The descriptive variables are expressed as the mean ± standard deviation if they followed a normal distribution after applying the Kolmogorov–Smirnov test, otherwise they are expressed as the median (interquartile range). Repeated measures ANOVA was used to compare continuous outcomes. One-to-one comparison was performed using repeated measures t-test or non-parametric Wilcoxon if normal distribution was ruled out. Statistical significance was set at *p* < 0.05. The statistical analysis was carried out using SPSS (SPSS Inc., Chicago, IL, USA) version 22.

## Results

### Baseline characteristics

Twenty-five patients with CKD stage 3–4, of whom 4 had a kidney transplant, were included. Table 1 shows the baseline characteristics. The mean age was 73.2 ± 5.9 years. The most usual aetiology of the heart failure was ischemic heart disease (56%), followed by dilated cardiomyopathy (16%). Sixty per-cent had received previous treatment with renin–angiotensin–aldosterone system (RAS) blockers, 95.8% with beta blockers, 73.9% with furosemide and 48% with a mineralocorticoid receptor (MCR) antagonist. The mean GFR was 29.4 ± 8.3 ml/min/1.73m^2^ and the LVEF 36.4 ± 8.9%.

**Table 1:** Baseline characteristics of the patients

**Variable**	***n* = 25**
Sex, male	21 (84)
Age, years	73.2 ± 5.9
Heart failure aetiology	
Ischaemic heart disease	14 (56)
Dilated cardiomyopathy	4 (16)
Other/Not known	7 (28)
LVEF, %	36.4 ± 8.9
Hypertension, yes	25 (100)
Systolic BP, mmHg	129.1 ± 20.3
Diastolic BP, mmHg	70.3 ± 14.9
Antihypertensive drugs, number	2 (1.5–3)
RAS blocker, yes	15 (60)
Beta-blocker, yes^a^	23 (95.8)
MRA, yes	11 (44)
Furosemide, yes^b^	17 (73.9)
Diabetes, yes	17 (68)
Dyslipidaemia, yes	23 (92)
Serum creatinine, mg/dL	2.2 ± 0.6
eGFR, mL/min/1.73m2	29.4 ± 8.3
Potassium, mEq/L	4.61 ± 0.6
PCR, mg/g	56.4 (26.3–427)
Visits to ED^c^, number	1 (0–2)

Values are given as n (%), mean ± SD, and median (IQR)

*Abbreviations*: *BP* Blood pressure, *ED* Emergency Department, *eGFR* Estimated glomerular filtration rate, calculated by CKD-EPI equation, *LVEF* Left ventricular ejection fraction, *MRA* Mineralocorticoid receptor antagonist, *PCR* urine protein/creatinine ratio; RAS: renin–angiotensin–aldosterone system

^a ^Only available in 24 patients

^b ^Only available in 23 patients

^c ^Due to congestive symptoms. Value represents those visits over one year prior to administration of sacubitril/valsartan

**Fig. 1:**
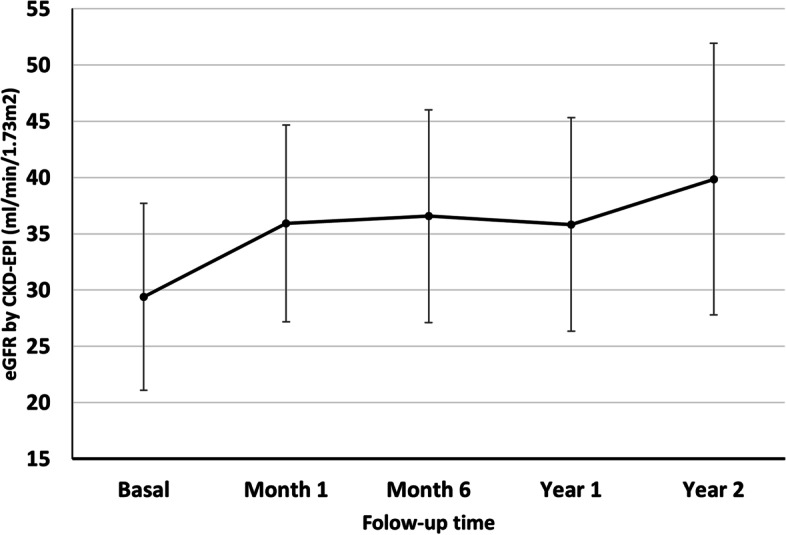
Evolution of renal function after administration of sacubitril/valsartan. Data are shown as mean ± SD. *p* = *0.019* (Repeated measure ANOVA)

**Fig. 2:**
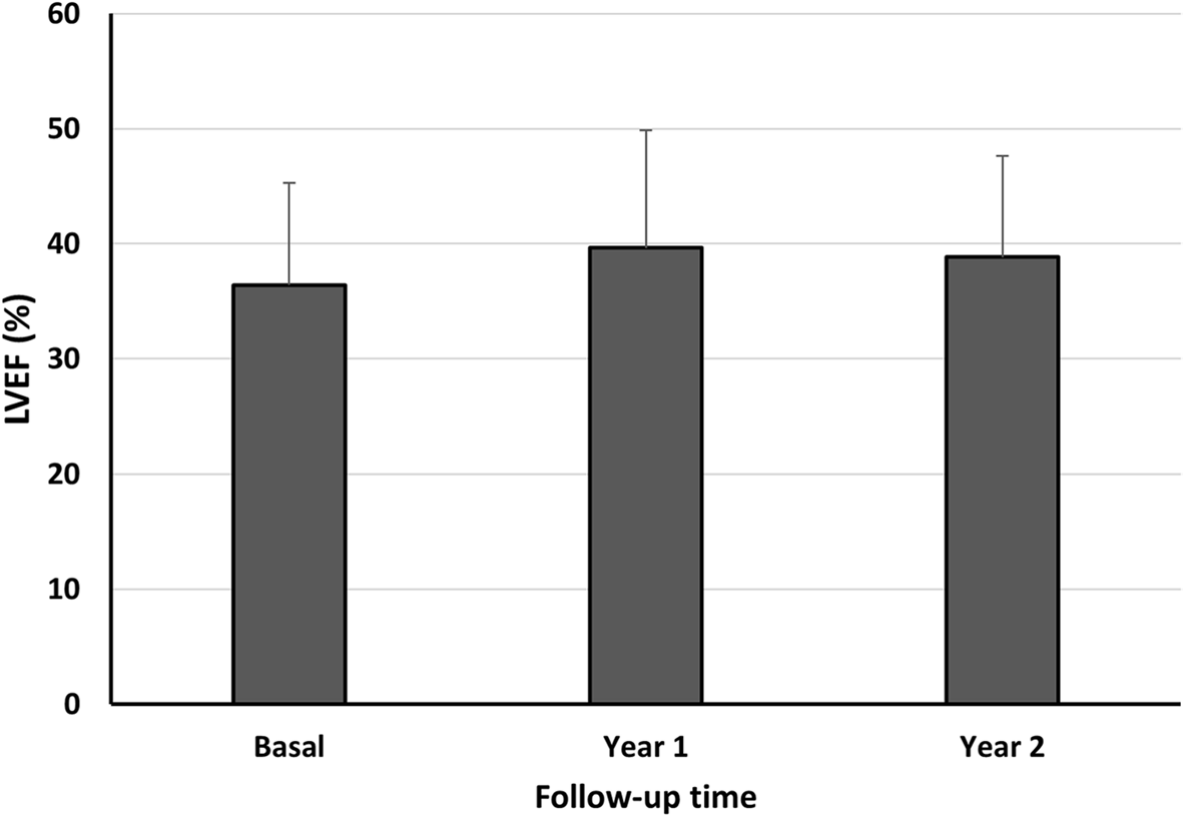
Evolution of left ventricular ejection fraction after administration of sacubitril/valsartan. Data are shown as mean ± SD. *p* = *0.018* (Repeated measure ANOVA)

The median follow-up period was 31 months (IQR 23.5–35). Characteristics at baseline and during treatment with sacubitril/valsartan are shown in Table 2. With effect from one month of treatment an improvement was noted in the GFR when compared by ANOVA (F = 3.396, *p* = 0.019) (Fig. 1). However, a post hoc analysis using the Bonferroni method was incapable of finding where these differences stemmed from. The increase in the GFR was accompanied by a significantly enhanced LVEF after the first year (*p* = 0.018) (Fig. 2), followed by a non-significant improvement in the subsequent years. The number of visits to the emergency department due to congestive symptoms fell throughout the follow-up compared with the year before starting the treatment, a change that was statistically significant in the second year (*p* = 0.045). Concerning non-pharmacological therapy, one patient received an implantable cardioverter-defibrillator (ICD) and another required cardiac resynchronization therapy (CRT). The remaining 23 patients needed no intervention after starting the treatment.

**Table 2:** Characteristics at baseline and during treatment with sacubitril/valsartan

	**Baseline *n* = 22**	**First year *n* = 17**	**Second year *n* = 13**
Serum creatinine, mg/dL	2.2 ± 0.6	1.9 ± 0.5	1.7 ± 0.4
eGFR, mL/min/1.73m2	29.4 ± 8.3	35.8 ± 9.4	39.8 ± 12.0
Potassium, mEq/L	4.61 ± 0.6	4.90 ± 0.6	4.87 ± 0.6
Urine protein:creatinine, mg/g	56.4 (26.3–427)	165 (100–313)	264 (51.3–1007.9)
Systolic blood pressure, mmHg	129.1 ± 20.2	124.2 ± 17.3	123.0 ± 18.1
Diastolic blood pressure, mmHg	70.2 ± 14.9	72.4 ± 11.0	70.4 ± 11.6
LVEF, %	36.4 ± 8.9	39.6 ± 10.2	38.8 ± 8.8
Visits to ED, number	1(0–2)	0 (0–0.3)	0 (0–0)

Recorded at scheduled visits with the nephrologist. Values are all given as mean ± SD and median (IQR)

*ED* Emergency Department, *eGFR* Estimated glomerular filtration rate calculated with CKD-EPI formula, *LVEF* Left ventricular ejection fraction

### Safety

No significant changes were seen in the figures for potassium or blood pressure. Although a 50% dose reduction of furosemide was applied at the start of the new treatment, none of our patients needed to increase the dose or initiate new diuretics afterwards. Only one patient discontinued the treatment (due to hypotension) and no symptoms suggestive of orthostatic hypotension were recorded. During the follow-up, 4 patients died (1 from cardiovascular causes, 1 from neoplasm, 1 from sepsis and 1 from post-surgical complications). The cardiovascular death occurred at 10 months of follow-up. No patient required renal replacement therapy.

## Discussion

This study undertaken in conditions of usual clinical practice shows that sacubitril/valsartan has a potentially beneficial role in the treatment of patients with advanced CKD and heart failure with a reduced LVEF, improving the GFR and ejection fraction after the first year of treatment.

Since the publication of PARADIGM other studies have appeared that also corroborate a lower reduction in the GFR with this drug [11–13]. However, clinical evidence was still lacking for patients with advanced CKD (stage 3b-4), who are commonly excluded from most clinical trials. Our study shows an improvement in renal function after initiating the treatment.

Initially, the beneficial role of sacubitril/valsartan on kidney function was thought to be attributable to improvement in the LVEF, though this failed to account for it completely. This drug can participate in other mechanisms involved in renal haemodynamics, such as vasoconstriction of the efferent arteriole, vasodilatation of the afferent arteriole and relaxation of the mesangial cells by the natriuretic peptides, factors that lead to an increased GFR [14].These haemodynamic mechanisms were firstly acknowledge as being a proteinuria enhancer, but now it seems remotely true. The UK HARP III trial included 79 (38%) patients with GFR less than 30 in the sacubitril/valsartan arm [15]. Comparatively, our study managed to include 15 patients with stage 4 CKD, representing 65% of the sample. While this trial showed similar effects on renal function and albuminuria between the sacubitril/valsartan and irbesartan groups, with no significant differences, in our study we found a better outcome on eGFR after initiating sacubitril/valsartan and removing former RAS inhibitors. Our findings suggest that sacubitril/valsartan somehow offers renoprotective qualities, which have been previously reported in a preclinical model of diabetes and hypertension, unrelated to antihypertensive efficacy, haemodynamic effects or inflammation, but that may be related to a protective effect of natriuretic peptides on podocyte integrity [16]. We speculate that the increase in protein excretion may be due to progression of diabetic kidney disease in our study. This aetiology was present in 57% of the sample that reached the 2-year follow-up. Moreover, no statistical difference was found when comparing proteinuria excretion throughout the follow-up.

CKD is associated with greater resistance to diuretics [17], which, in turn, is independently associated with overall mortality, sudden death and death due to pump failure [14]. Sacubitril/valsartan seems to provide a relevant benefit, derived from inhibition of the degradation of natriuretic peptides by its action on the proximal and distal tubules, increasing excretion of sodium and water. In our cohort we noted fewer emergency visits due to congestive symptoms, despite reducing the dose of the loop diuretic at the same time as the treatment was started.

In general, patients with heart failure and reduced LVEF present an anomalous cardiac remodelling (CR). CR is a process in which molecular, genetic, cellular and interstitial changes occur that lead to left ventricular dysfunction after having experienced a myocardial lesion or important myocardial stress. Patients with major CR show progressive worsening of cardiac function, resulting in a considerable increase in cardiovascular morbidity and mortality [18]. Accordingly, avoiding progressive CR requires establishing an optimal early medical treatment strategy with drugs or devices.

The PROVE-HF and EVALUATE-HF studies confirmed the positive impact of sacubitril/valsartan on CR in patients with heart failure and a reduced LVEF [19, 20]. Likewise, the meta-analysis by Wang showed that NEPi acted rapidly, with more pronounced changes over time, concluding that patients could benefit from early treatment with a NEPi for at least 3 months [21]. In our study we saw a significant increase in LVEF at one year of treatment as compared to baseline. The positive trend ceased to be significant during the two years after follow-up, which could be explained by the small sample and the irregular follow-up in some cases.

Of the 25 patients in the study, only two were candidates for ICD and CRT after starting sacubitril/valsartan, both during the first month of treatment; the effect probably being marginal on the overall analysis. In fact, the results are compatible with the clinical evidence accumulated so far that angiotensin receptor-neprilysin inhibition is beneficial, even with effect from the first week of treatment, regarding quality of life, progression of the heart disease and survival, as well as in the improvement in biomarkers and CR [5, 18, 19, 22–24].

A feared effect of sacubitril/valsartan in advanced CKD is hyperkalaemia. Although 44% of the patients in our cohort were taking a mineralocorticoid receptor antagonist (MRA) at the start of the study, no cases of hyperkalaemia were seen in any of those who continued this treatment. A subanalysis of the PARADIGM-HF study evaluated the incidence of hyperkalaemia in patients with symptomatic heart failure and reduced ejection fraction who were being treated with MRA and sacubitril/valsartan versus enalapril [25]. Overall, the incidence of hyperkalaemia was low and similar between groups, although there was a greater incidence of severe hyperkalaemia in the enalapril group among those patients who had been using MRA beforehand. We can conclude that the precautions in the titration of sacubitril/valsartan do not differ significantly from what occurs with the titration of ACEI, in which even more caution should be exercised.

Together with hyperkalaemia, hypotension is the most usual adverse effect noted in the clinical trials. In our cohort, only one patient required treatment withdrawal for this reason.

It is notable that, despite the high cardiovascular mortality in patients with advanced CKD and heart failure with reduced LVEF, only one of our patients died from this cause during the two years of follow-up. The benefits of this drug on long-term survival seen in our study should be assessed in other suitable studies.

The most notable limitations of our study concern the small number of patients recruited and its retrospective nature. Nonetheless, the clinical follow-up was very close over a long period of time. Finally, we are unable to establish any cause-effect relationship in our analysis due to the nature of our observational study.

## Conclusions

In conclusion, sacubitril/valsartan could be administered safely as a first-line drug, given the benefits noted on renal function, LVEF and the control of congestive symptoms in our patients with advanced CKD and reduced LVEF.

## Data Availability

Data are available on request due to privacy restrictions. The data presented in this study are available on request from the corresponding author. In compliance with Spanish Organic Law 15/1999, the data are not publicly available.

## Abbreviations

ACEI: Angiotensin converting enzyme inhibitors
ARAII: Angiotensin receptor antagonist II
CKD: Chronic kidney disease
CR: Cardiac remodelling
CRT: Cardiac resynchronization therapy
GFR: Glomerular filtration rate
HF: Heart failure
ICD: Implantable cardioverter-defibrillator
LVEF: Left ventricular ejection fraction
MCR: Mineralocorticoid receptor
NEPi: Neprilysin inhibitor
RAS: Renin angiotensin system
